# A Versatile Solid-Phase Approach to the Synthesis of Oligonucleotide Conjugates with Biodegradable Hydrazone Linker

**DOI:** 10.3390/molecules26082119

**Published:** 2021-04-07

**Authors:** Mariya I. Meschaninova, Nina S. Entelis, Elena L. Chernolovskaya, Alya G. Venyaminova

**Affiliations:** 1 Institute of Chemical Biology and Fundamental Medicine, Siberian Branch of Russian Academy of Sciences, 630090 Novosibirsk, Russia; elena_ch@niboch.nsc.ru (E.L.C.); ven@niboch.nsc.ru (A.G.V.); 2 UMR Genetique Moleculaire, Genomique, Microbiologie (GMGM), Strasbourg University—CNRS, 67084 Strasbourg, France; n.entelis@unistra.fr

**Keywords:** solid-phase 5′-functionalization, lipophilic conjugates of oligonucleotides, pH-sensitive hydrazone covalent bonds, siRNA, mitochondrial antireplicative and guide RNAs

## Abstract

One of the ways to efficiently deliver various drugs, including therapeutic nucleic acids, into the cells is conjugating them with different transport ligands via labile or stable bonds. A convenient solid-phase approach for the synthesis of 5′-conjugates of oligonucleotides with biodegradable pH-sensitive hydrazone covalent bonds is proposed in this article. The approach relies on introducing a hydrazide of the ligand under aqueous/organic media to a fully protected support-bound oligonucleotide containing aldehyde function at the 5′-end. We demonstrated the proof-of-principle of this approach by synthesizing 5′-lipophilic (e.g., cholesterol and α-tocopherol) conjugates of modified siRNA and non-coding RNAs imported into mitochondria (antireplicative RNAs and guide RNAs for Mito-CRISPR/system). The developed method has the potential to be extended for the synthesis of pH-sensitive conjugates of oligonucleotides of different types (ribo-, deoxyribo-, 2′-*O*-methylribo-, and others) with ligands of different nature.

## 1. Introduction

Synthetic RNA and DNA (antisense oligonucleotides, catalytic NAs, siRNAs, antagomirs, miRNA mimics, aptamers, guide RNAs of the CRISPR system, and others) and constructs on their basis are now of huge interest as promising therapeutic and diagnostic agents (see, e.g., [1,2,3] and references therein). Important issues in this field are the efficiency of cellular uptake of oligonucleotides and the preciseness of their subsequent delivery to the particular site of their action in the cytosol or nucleus of tissue cells. One of the ways to address these problems is to create oligonucleotide conjugates with different ligands ([4,5,6] and references therein). Among them, there could be delivery agents, e.g., receptor-specific or improving transmembrane penetration. For this purpose, one can use peptides and proteins, carbohydrates, polyamines, lipophilic compounds, small molecules, NA aptamers, nanoparticles, and others (see, e.g., [7,8,9,10,11,12,13,14,15,16]).

To synthesize the oligonucleotide conjugates, it is possible to use two general synthetic strategies—solution-phase and solid-phase approaches, each with its own benefits and drawbacks (see, e.g., [4,17,18] and references therein).

Usually, ligands are attached to oligonucleotides via different linkers, and both the length and nature of these linkers determine to a certain extent the biological effects of the conjugates. The capability of chemical bonds connecting two molecular entities of degrading inside the cell with the release of the oligonucleotide from the conjugate allows for the design of prospective drugs and diagnostic tools. A number of labile linkers adapted for different cleavage conditions by enzymes, nucleophilic/basic or electrophilic/acidic reagents, reducing or oxidizing agents, photo-irradiation, organometallic and metal reagents have been suggested (see, e.g., [19,20,21,22,23,24,25]). Chemical conjugation of therapeutic NA through acid-sensitive bonds (acetal, ketal, hydrazone, β-thiopropionate, and others (see, e.g., [18,20])) prevents the destruction of oligonucleotide conjugates in the bloodstream (pH 7.4) and thereby protects normal healthy cells from the adverse effects of therapeutic drugs. After entering the cell, the acid-labile bond degrades in compartments of the endo/lysosomal pathway (pH 5.0–6.0) [26], setting the therapeutic NA free for its specific action without any hindrances from the delivery ligand.

In general, pH-sensitive conjugates of oligonucleotides containing a hydrazone bond are synthesized through the interaction between fully unprotected aldehyde-containing oligonucleotides and hydrazides of small molecules, antibodies, peptides, proteins, and others (see, e.g., [27,28,29]). To that end, oligonucleotides with aldehyde group at various positions of the chain (5′- or 3′-end, heterocyclic base as well as 1′- or 2′-position of ribose) are obtained by several ways: (1) via 1,2-diols followed by oxidation with periodate (see, e.g., [30,31,32,33]); (2) by synthesizing oligonucleotides with an aldehyde group protected with substituted 1,3-propanediols, which are removed during mild acid treatment (see, e.g., [34,35,36]); (3) using oligonucleotides with masked AP-site (see, e.g., [37,38,39]); (4) through special aldehyde-succinimide modifiers (see, e.g., [40,41,42]). Much less commonly, the reverse order of interaction is exploited: ligands carrying an aldehyde function interact with unprotected hydrazide-containing oligonucleotides (see, e.g., [43,44]).

Of note, all mentioned above approaches to the synthesis of pH-labile hydrazone containing oligonucleotide conjugates are based on solution-phase strategies. In the given work, we developed the convenient solid-phase approach to the synthesis of 5′-conjugates of oligonucleotides containing pH-sensitive hydrazone bonds.

## 2. Results

**Figure 1:**
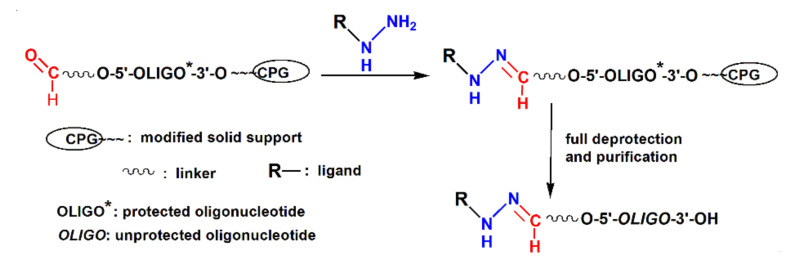
The general scheme of the solid-phase synthesis of 5′-conjugates of oligonucleotides with hydrazone bond.

Here, we describe the solid-phase approach to the synthesis of oligonucleotides conjugated with delivery agents through hydrazone bonds, based on the addition of hydrazides of delivery ligands to the 5′-aldehyde-modified protected oligonucleotides attached to the solid support (Figure 1).

We demonstrated the proof-of-concept of our approach on a range of conjugates of model oligodeoxyribonucleotide and functional RNAs and their stability in biological media 2′-*O*-methyl analogs, which will be further employed for biological studies in our subsequent works. Therapeutic RNAs are represented in this work by siRNA targeting *MDR1* mRNA and non-coding RNAs imported into mitochondria (antireplicative RNAs and guide RNAs for Mito-CRISPR/system) (see Table 1 and references therein). As examples of delivery agents, we selected cholesterol and α-tocopherol, well-known lipophilic transport ligands improving pharmacokinetic and cellular uptake properties of therapeutic nucleic acids (see, e.g., [12,45,46]).

### 2.1. Synthesis of Hydrazides of Lipophilic Compounds (***VIII*** and ***IX***)

**Figure 2:**
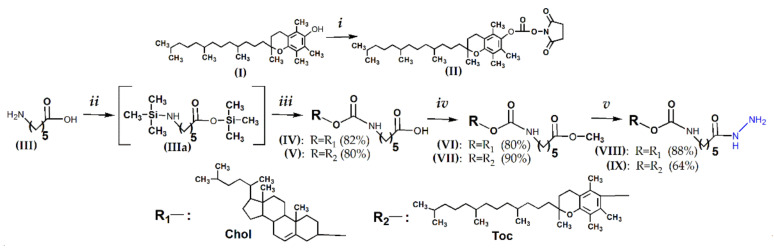
General scheme of the synthesis of hydrazides of cholesterol (**VIII**) and α-tocopherol (**IX**). Reagents: (***i***) DSC, CH_2_Cl_2_, CH_3_CN, triethylamine (TEA), room temperature (RT); (***ii***) (CH_3_)_3_SiCl, pyridine (Py), 4 °C; (***iii***) CholOC(O)Cl or *N*-succinimide derivative of α-tocopherol (**II**), Py, RT; 0.7 M HCl; (***iv***) PCl_3_, CH_2_Cl_2_, argon, RT; CH_3_OH abs, RT; and (***v***) NH_2_NH_2_^•^H_2_O, CH_3_OH, RT.

According to the proposed solid-phase approach, first, we synthesized the hydrazides of cholesterol (**VIII**) and α-tocopherol (**IX**) (see the synthetic scheme in Figure 2). Commercially available cholesterol chloroformate and *N*-succinimide derivative of α-tocopherol (**II**) obtained using *N*,*N*’-disuccinimidyl carbonate (DSC) [14] (Figure 2, step *i*) were taken as initial activated derivatives of cholesterol and α-tocopherol. Their interaction with 6-aminohexanoic acid (**III**) via *O*,*N*-*bis*-trimethylsilyl intermediate (**IIIa**) [47] gave cholesterol or α-tocopherol derivatives of trimethylsilyl ester of 6-aminohexanoic acid, easily transformed under acidic conditions into compounds (**IV**) and (**V**) (Figure 2, steps *ii* and *iii*). The carboxy groups of (**IV**) and (**V**) were converted to acyl chlorides by the action of phosphorus trichloride, followed by conversion to methyl esters by the action of methanol (Figure 2, step *iv*). The resulting derivatives (**VI**) and (**VII**) interacted with hydrazine monohydrate, giving the desired cholesterol (**VIII**) and α-tocopherol (**IX**) hydrazides (Figure 2, step *v*). All reactions were characterized by high yields. It is worth noting that the hydrazide of α-tocopherol was obtained for the first time in our work. The structures of the compounds (**IV)**, (**VI**), and (**VIII**) were confirmed by ^1^H-NMR [48] (Supplementary Figures S1, S3 and S5). The structures of the compounds (**V**), (**VII**), and (**IX**) were confirmed by ^1^H-NMR, ^13^C-NMR, and ESI-MS (Supplementary Figures S2, S4 and S6).

### 2.2. Solid-Phase Synthesis of Lipophilic Conjugates of Oligonucleotides

Heptadeoxyribonucleotide (dT_7_) for model experiments and functional oligoribonucleotides (sense strand of siRNA targeted *MDR1* mRNA, mitochondrial antireplicative RNAs and guide RNAs for Mito-CRISPR/system) partially modified by the introduction of 2′-*O*-methyl ribonucleotides to enhance their nucleolytic stability were synthesized by the phosphoramidite method on an automatic synthesizer ASM-800 (Biosset, Novosibirsk, Russia) using deoxyribo-, ribo-, and 2′-*O*-methylribo-phosphoramidites with ultra-mild base protecting groups. At the last synthetic cycle, we used a non-nucleotide 5′-Aldehyde-modifier C2 phosphoramidite (Glen Research) containing a 4-(2-hydroxyethoxy)benzaldehyde residue protected with 2,2-dimethylpropane-1,3-diol. Therefore, the masked aldehyde group was introduced at the 5′-end of the oligonucleotide chain, giving fully protected aldehyde-containing polymer-bound oligonucleotides.

In advance, we optimized the conditions of hydrazone bond formation during the solid-phase synthesis of conjugates. We also verified its stability in the course of deblocking conjugates using a series of model experiments (see Section 4.4, Supplementary Figures S7 and S8).

**Figure 3:**
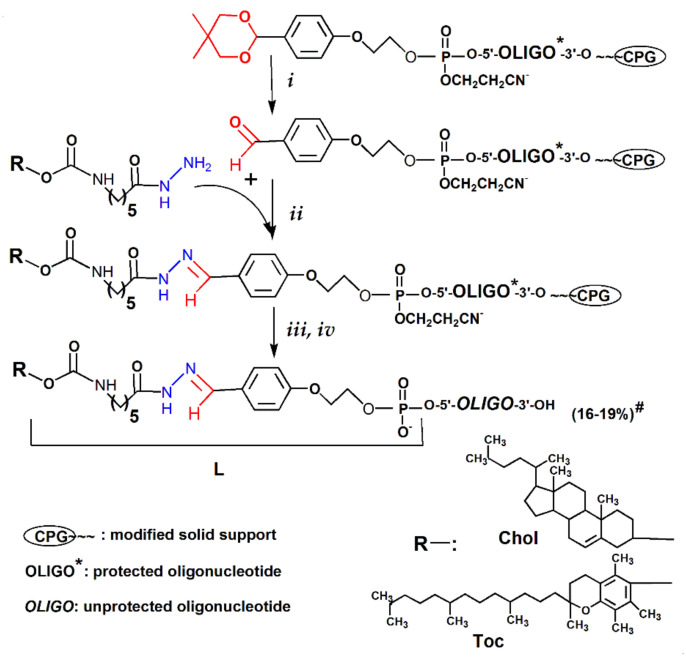
General scheme of the synthesis of 5′-lipophilic conjugates of oligonucleotides. Reagents: (***i***) 80% acetic acid, 1 h, RT; (***ii***) 0.1 M NaOAc(pH 5.2)/dioxane (1/1), 12 h, RT; (***iii***) 0.05 M K_2_CO_3_ in methanol, 16 h, RT; and (***iv***) NMP/TEA^•^3HF/TEA (150/100/75), 1.5 h, 65 °C. Sequences of oligonucleotides are shown in Table 1. L: -O-C(O)NH-(CH_2_)_5_-C(O)NH-N=CH-C_6_H_4_-O-(CH_2_)_2_-O-P(O)(OH)-; Chol: cholesterol residue; Toc: α-tocopherol residue. (#) The yields of conjugates after deblocking and isolation by denaturing PAGE were calculated relative to the first nucleoside on the polymer support.

The lipophilic oligoribonucleotide conjugates (**2**–**17**) were obtained under optimized conditions according to the scheme shown in Figure 3. Protected aldehyde-modified polymer-bound oligoribonucleotides were treated with 80% acetic acid and then with the mixture of 0.1 M NaOAc (pH 5.2) with the same volume of hydrazides of lipophilic ligands in dioxane. After that, the oligonucleotides were subjected to ultra-mild deprotection with 0.05 M K_2_CO_3_ in methanol, and removal of 2′-*O*-protected groups with *N-*methyl-2-pyrrolidinone(NMP)/TEA^•^3HF/TEA was done. The reverse-phase HPLC of the reaction mixtures on the ProntoSIL-120-5-C18 AQ column (EcoNova, Novosibirsk, Russia) (gradient elution from 0 to 50% (25 min) of acetonitrile in 0.02 M triethylammonium acetate buffer, pH 7.0) led to a significant loss of hydrophobic conjugates (up to 70%) regardless of the oligonucleotide length. Therefore, we used denaturing PAGE under optimized conditions for analysis and purification of the conjugates. Structures of conjugates are given in Table 1. We also demonstrated by the example of conjugates (**5**) and (**7**) that the treatment of a polymer-bound protected conjugate with sodium borohydride easily and quickly reduces a labile hydrazone bond to a stable hydrazine one.

**Table 1:** Synthesized conjugates of oligonucleotides.

N	Conjugate	Sequence, 5′ → 3′ ^1^	Type of Oligonucleotide
**1**	Chol-l-dT_7_ (7nt)	d(TTTTTTT)	Model oligodeoxyribonucleotide
**2**	Chol-l-siRNA/s (21nt)	Chol-l-GGCUU^m^GAC^m^AAGUU^m^GU^m^AU^m^AU^m^GG	siRNA ^2^
**3**	Toc-l-siRNA/s (21nt)	Toc-l-GGCUU^m^GAC^m^AAGUU^m^GU^m^AU^m^AU^m^GG	
**4**	Chol-l-C57/1 (40nt)	Chol-l-CAAAUUUUAACUCUCCAAACGAGCCCCC UACAGGGCUCUU	Mitochondrial antireplicative RNAs ^3^
**5**	Chol-l_1_-C57/1 (40nt)	Chol-l_1_-CAAAUUUUAACUCUCCAAACGAGCCCCC UACAGGGCUCUU ^6^	
**6**	Chol-l-C57/2 (56nt)	Chol-l-GCGCAAUCGGUAGCGCCAAAUUUUAACUC UCCAAACGAGCCCCCUACAGGGCUCUU	
**7**	Chol-l_1_-C57/2 (56nt)	Chol-l_1_-GCGCAAUCGGUAGCGCCAAAUUUUAACUC UCCAAACGAGCCCCCUACAGGGCUCUU ^6^	
**8**	Chol-l-C57/3 (56nt)	Chol-l-GCGC^m^AAUC^m^GGU^m^AGCGCCAAAUUUU AACUCUCCAAACGAGCCCCCU^m^AC^m^AGGGCUCUU	
**9**	Chol-l-C57/4 (56nt)	Chol-l-GCGC^m^AAUC^m^GGU^m^AGCGCGUUU GGAGAGUUAAAAUUUGGAGCCCCCU^m^AC^m^AGGGCUCUU	
**10**	Chol-l-HB/1 (56nt)	Chol-l-GCGCA^m^AUC^m^GGU^m^AGCGCAUCUUAACUCUC UUUUAACUGAGCCCCCU^m^AC^m^AGGGCUCUU	
**11**	Chol-l-HB/2 (56nt)	Chol-l-GCGC^m^AAUC^m^GGU^m^AGCGCAGUUAAAA GAGAGUUAAGAUGAGCCCCCU^m^AC^m^AGGGCUCUU	
**12**	Chol-l-KSS/1 (62nt)	Chol-l-GAGAAGUAAGCACUGUAAAGGUUUU AGAGCUAGAAAUAGCAAGUUAAAAUAAGGCUAGUCCG	sgRNAs ^4^
**13**	Chol-l-KSS/2 (84nt)	Chol-l-GAGAAGUAAGCACUGUAAAGGUUUU AGAGCUAGAAAUAGCAAGUUAAAAUAAGGCUAGUCCG UUGAGCCCCCUACAGGGCUCUU	
**14**	Chol-l-crRNA/1 (40nt)	Chol-l-UAAUUUCUACUCUUGUAGAUGAU GAUGUGGUCUUUGGAGU	crRNAs ^5^
**15**	Toc-l-crRNA/1 (40nt)	Toc-l-UAAUUUCUACUCUUGUAGAUGAU GAUGUGGUCUUUGGAGU	
**16**	Toc-l-crRNA/2 (40nt)	Toc-l-UAAUUUCUACUCUUGUAGAUGAU GAUGUGGUUUUUGGAGU	
**17**	Toc-l-crRNA/3 (40nt)	Toc-l-UAAUUUCUACUCUUGUAGAUGAU GAUGUGGCCUUUGGAGU	

^1^ dN: deoxyribonucleotide; N: ribonucleotide; N^m^: 2′-*O*-methyl-ribonucleotide. ^2^ Sense strand of siRNA, corresponding to the 557–577 nt region of *MDR1* mRNA [49]. ^3^ Antireplicative RNAs [50] targeting control region of mouse mtDNA, haplotypes HB and C57BL/6N [51]. ^4^ Guide RNAs recognizing the border of the KSS deletion, m.8363-15438del mtDNA [52]. ^5^ crRNAs for CRISPR/Cas12a system targeting human mtDNA [53]. ^6^ Conjugate containing reduced hydrazone bond. Chol: cholesterol residue; Toc: α-tocopherol residue; L: -O-C(O)NH-(CH_2_)_5_-C(O)NH-N=CH-C_6_H_4_-O-(CH_2_)_2_-O-P(O)(OH)-; L_1_: -O-C(O)NH-(CH_2_)_5_-C(O)NH-NH-CH_2_-C_6_H_4_-O-(CH_2_)_2_-O-P(O)(OH)-.

Synthesized conjugates (**2**, **3**, **5**, **7**) were characterized by ESI-MS analysis (Supplementary Figures S10–S14). At the ESI mass spectrum of Chol-l-siRNA/s (**2**) and Toc-l-siRNA/s (**3**), which were homogeneous according to gel electrophoresis, in addition to peaks corresponding to target conjugate (**2**) or (**3**), we also observed the peaks related to the original aldehyde-containing oligonucleotide Ald-siRNA/s. This phenomenon points to the destruction of the hydrazone bond during mass analysis (Supplementary Figures S11 and S12). In the case of the Toc-l-siRNA/s conjugate (**3**), in addition to the peaks related to Toc-l-siRNA/s and Ald-siRNA/s, the third series of peaks related to the isocyanate derivative of siRNA/s formed after the α-tocopherol residue tear-off was recorded, which is consistent with the literature data [54] (Supplementary Figure S12). When the number of nucleotides in an oligonucleotide chain increased from 21 to 84nt, the resolution of the spectra dramatically decreased. One of the ways to partially solve this problem is the reduction in the labile hydrazone bond in conjugates and its conversion into stable hydrazine bond, as it was demonstrated by us on examples of ESI-MS spectra of Chol-L_1_-C57/1 (**5**) and Chol-L_1_-C57/2 (**7**) (Supplementary Figures S13 and S14).

### 2.3. Stability of the Lipophilic Conjugates at Different pH

**Figure 4:**
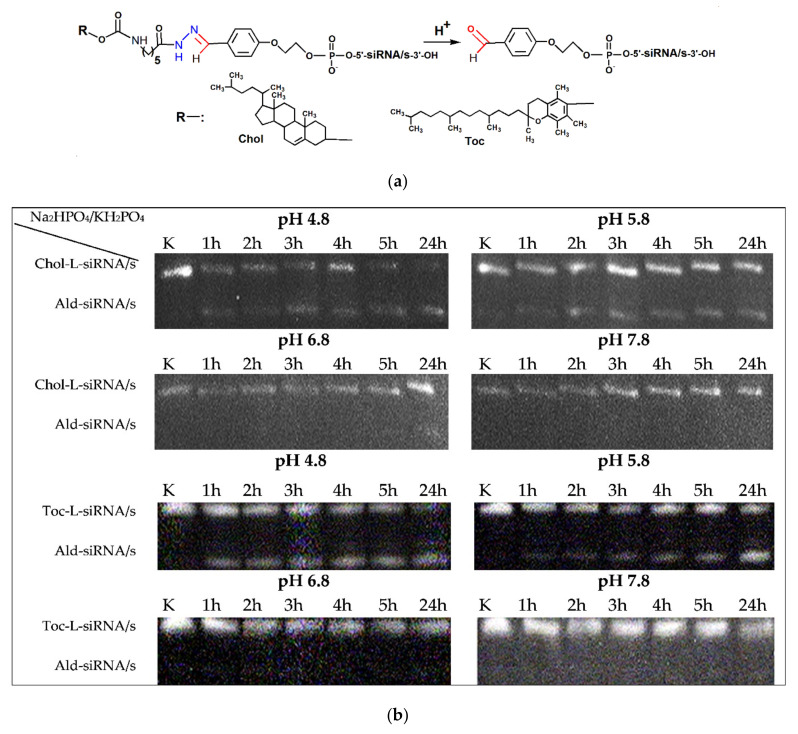
Cleavage of the hydrazone bond at Chol-l-siRNA/s (**2**) and Toc-l-siRNA/s (**3**) conjugates. (**a**) Common structure of lipophilic conjugates and aldehyde derivative of siRNA/s and (**b**) stability of lipophilic conjugates upon incubation in phosphate buffer at different pHs. K: (**2**) or (**3**); Chol: cholesterol residue; Toc: α-tocopherol residue; Ald: C(O)H-C_6_H_4_-O-(CH_2_)_2_-O-P(O)(OH)-; L: -O-C(O)NH-(CH_2_)_5_-C(O)NH-N=CH-C_6_H_4_-O-(CH_2_)_2_-O-P(O)(OH)-.

The comparative study of influence of pH and lipophilic ligand type on stability of hydrazone bond was done by the examples of conjugates of siRNA sense strand bearing cholesterol or α-tocopherol residues (Figure 4). For this purpose, Chol-l-siRNA/s (**2**) or Toc-l-siRNA/s (**3**) conjugates (1 *A*_260_ unit) were incubated for 1, 2, 3, 4, 5, and 24 h at 37 °C in Na_2_HPO_4_/KH_2_PO_4_ buffer at pH 4.8, 5.8, 6.8, or 7.8. Reaction products were analyzed by denaturing 12% PAGE and stained with ethidium bromide. It was shown that at pH values close to neutral, these conjugates remain stable. In contrast, most part of the both conjugates was hydrolyzed at pH 4.8, which is close to pH in endosomes. It can also be concluded that, in general, the α-tocopherol conjugate is more stable than cholesterol conjugate.

## 3. Discussion

In the present work, we proposed a novel method of solid-phase synthesis of oligonucleotide conjugates with stimuli-sensitive hydrazone bond. The approach is based on the attachment of an aldehyde group to the 5′-end of protected polymer-bound oligonucleotide obtained by an automated oligonucleotide synthesis using special phosphoramidite at the last synthetic cycle, followed by the interaction with hydrazide-containing ligands and ultra-mild deprotection (Figure 3).

The proof-of-principle of this approach was demonstrated by the synthesis of 5′-lipophilic conjugates of siRNA, mitochondrial antireplicative RNAs, and guide RNAs for the Mito-CRISPR system. As lipophilic ligands, we used cholesterol and α-tocopherol, which can interact with transmembrane receptors, lipoproteins, and other blood proteins (see, e.g., [7]).

It should be noted that hydrazone bond can be obtained from aldehydes or ketones and hydrazides under mild conditions using a wide range of solvents, such as benzene, esters, DMF, DMSO, alcohols, as well as aqueous solutions (see, e.g., [55]). Kratz et al. studied the hydrolytic stability of hydrazones on a series of model compounds (see, e.g., [56]). According to these data, the use of aromatic aldehyde as a carbonyl component and aliphatic hydrazide for the synthesis of hydrazone is optimal. It provides relative stability of the compound itself with a high hydrolysis rate of hydrazone bond under acidic conditions. It was suggested that two main factors affect the efficiency of adsorption of conjugates on cell surface: hydrophobicity of conjugates and the distance between negatively charged cell membrane and anionic RNA. Taking this into account, in the present work, we used a long aliphatic linker containing 2 carbon atoms, hydrazone bridge, and another 6 carbon atoms between 5′-end of RNA and lipophilic ligand to provide an optimal distance between the cell membrane and RNA part.

For this purpose, we modified lipophilic ligands with aminohexanoic acid followed by converting its carboxyl group to hydrazide. The hydrazides of cholesterol (**VIII**) and α-tocopherol (**IX**) were obtained in four steps with good yields (Figure 2). The synthesis of oligoribonucleotides and introducing of the masked aromatic aldehyde group at the 5′-end were carried out by an automatic phosphoramidite method.

Going to the solid-phase synthesis of 5′-conjugates of oligonucleotides with hydrazone linkage, we started from the model experiments. We investigated the coupling of a ligand with an aldehyde-modified support-bound oligonucleotide and the stability of the formed hydrazone bond under the deblocking conditions. In the works of Oretskaya et al. devoted to the synthesis and use of aldehyde derivatives of oligonucleotides to create the conjugates of various structures and stability, the authors note the instability of hydrazone bond under basic conditions, including ammonia treatment (see, e.g., [31,32,57,58]). Our model studies on the 5′-cholesterol-dT_7_ conjugate with hydrazone bond (Chol-l-dT_7_) allowed for selecting deblocking conditions for the synthesized conjugates, which preserve the hydrazone bond from breaking down. It was shown that the hydrazone bond at Chol-l-dT_7_ is stable at processing with TEA^•^3HF as well as in the presence of 0.05 M K_2_CO_3_ in methanol (see Section 4.4 and Supplementary Figure S8). In all other basic conditions, we observed degradation of the synthesized conjugate to the initial aldehyde-containing oligonucleotide.

While selecting the optimal conditions for the solid-phase synthesis of hydrazone-containing Chol-l-dT_7_, we found that a major role belongs to the ratio of aqueous and organic phases (Supplementary Figure S7).

The model optimization experiments allow us to obtain the series of lipophilic conjugates of RNAs by the interaction of the hydrazide of cholesterol (**VIII**) or α-tocopherol (**IX**) with 5′-aldehyde modified polymer-bound protected oligonucleotide followed by complete deblocking and PAGE isolation (Figure 3). The yields of conjugates for all variants were 16–19% relative to the first nucleoside on the polymer support, which is compatible with the yields of synthesis of common non-modified oligoribonucleotides. The proposed method also permits obtaining conjugates with a stable bond between ligand and oligonucleotide by reducing on solid-phase the hydrazone bond under mild conditions.

The lability of hydrazone bond at different pHs was tested by an example of siRNA conjugates with cholesterol and α-tocopherol, namely, Chol-l-siRNA/s (**2**) and Toc-l-siRNA/s (**3**), incubated in phosphate buffer at pH 4.8–7.8.

**Figure 5:**
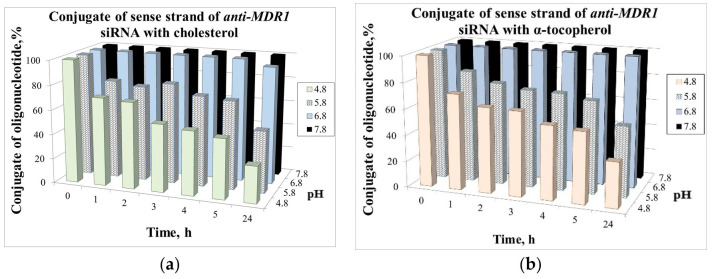
Hydrazone bond hydrolysis in Chol-l-siRNA/s (**a**) and Toc-l-siRNA/s (**b**) conjugates: quantification of the full-size conjugate (%, axis Y) depending on pH and the time of incubation.

We have shown that at pH 6.8–7.8, the conjugates remain stable. At pH 5.8, 50% of conjugates were hydrolyzed after 24 h of incubation, while at pH 4.8, ~50% were hydrolyzed after 5 h and ~70% after 24 h (Figure 4 and Figure 5). It can also be concluded that, in general, tocopherol conjugate is more stable than cholesterol conjugate.

Using this newly developed convenient solid-phase approach to the synthesis of lipophilic conjugates of NA with pH-triggered hydrazone bond, we synthesized the series of functional RNA conjugates for our future biological investigations.

## 4. Materials and Methods

### 4.1. General Information

Briefly, 5′-Aldehyde-Modifier C2-phosphoramidite, all phosphoramidites, and nucleoside-containing solid supports with base ultra-mild protecting groups for oligonucleotide synthesis were obtained from Glen Research (Sterling, VA, USA); 2,5,7,6-tetramethyl-2-(4′,8′,12′-trimethyltridecyl)-chroman-6-ol (α-tocopherol), cholesteryl chloroformate, *N*,*N*’-disuccinimidyl carbonate (DSC), 6-aminohexanoic acid, and silica gel for column chromatography (230–400 mesh, 60 Å) were purchased from Sigma-Aldrich (St. Louis, MO, USA); and hydrazine hydrate was obtained from Fluka (St. Louis, MO, USA). Other chemicals were supplied by Merck (Kenilworth, NJ, USA), Acros Organics (Geel, Belgium), and TCI Chemicals (Chennai, India). Solvents were supplied by Panreac (Barcelona, Spain) and dried by 3 Å molecular sieves or by distillation and stored over CaH_2_. Products were visualized on TLC plates (DC-Alufolien Kieselgel 60 F254, Merck, Darmstadt, Germany) at 254 nm ultraviolet light.

NMR spectra of the compounds were measured with CDCl_3_ as a solvent using AVANCE III 400 and 500 NMR spectrometers (Bruker Corporation, Billerica, MA, USA).

Mass spectra were recorded by the ESI LC/MS XCT (Agilent Technologies, Santa Clara, CA, USA).

The optical densities of the solutions of oligonucleotide conjugates were measured using a NanoDrop 1000 spectrophotometer (Thermo Fisher Scientific, Waltham, MA, USA).

Oligonucleotide products were analyzed by 15 or 20% PAGE (AA/bisAA 30:1, 7 M urea, TBE) and purified by 12% PAGE (AA/bisAA 30:0.5, 7 M urea, TBE). The gels after analytical gel-electrophoresis were either stained with ethidium bromide and quantified using “Gel Imager 2” (Helicon, Moscow, Russia) or stained with Stains-all dye for qualitative visualization.

### 4.2. Synthesis of Hydrazides of Lipophilic Compounds (***VIII***) and (***IX***)

The synthesis of hydrazide of 6-(cholesteryloxycarbonylamino)-hexanoate was fulfilled according to [48].

Briefly, 6-[2,5,7,6-Tetramethyl-2-(4′,8′,12′-trimethyltridecyl)-chroman-6-ol succinimidyl carbonate (**II**) was synthesized in accordance with [14] (Figure 2, step *i*). For this, 2,5,7,6-Tetramethyl-2-(4′,8′,12′-trimethyltridecyl)-chroman-6-ol (**I**) (1.1 g, 2.5 mmol) was dissolved in dry dichloromethane (10 mL), then TEA (1 mL, 7.5 mmol) and DSC (1 g, 3.9 mmol) in 5 mL acetonitrile were subsequently added, and the reaction mixture was stirred for 16 h under argon at RT. The reaction was monitored by TLC (5% C_2_H_5_OH in CH_2_Cl_2_). The reaction mixture was evaporated under reduced pressure; the residue was dissolved in dichloromethane (20 mL) and washed by saturated aqueous NaHCO_3_ (2 × 20 mL). The organic phase was dried under anhydrous Na_2_SO_4_ and evaporated under reduced pressure. Dry residue of (**II**) (1.4 g) was used without further purification.

6-(Cholesteryloxycarbonylamino)-hexanoic acid (**IV**) and 6-[2,5,7,6-Tetramethyl-2-(4′,8′,12′- trimethyltridecyl)-chroman-6-yloxycarbonyl]-hexanoic acid (**V**) (Figure 2, steps *ii*, *iii*). Briefly 6-Aminohexanoic acid (**III**) (1 g, 7.6 mmol) was suspended in dry pyridine (15 mL), and then, chlorotrimethylsilane (3.8 mL, 30 mmol) was added dropwise at 0 °C with the formation of intermediate (**IIIa**) by analogy with [47]. The mixture was stirred until the solution became clear, then cholesteryl chloroformate (1.1 g, 2.5 mmol) or compound (**II**) (1.4 g, 2.5 mmol) was added, and the reaction mixture was stirred for 3 h at RT. The reaction was monitored by TLC (5% C_2_H_5_OH in CH_2_Cl_2_). Pyridine was evaporated under reduced pressure; the residue was dissolved in dichloromethane (100 mL) and then washed with 0.7 M HCl (50 mL) and saturated aqueous NaCl (50 mL). The organic phase was dried under anhydrous Na_2_SO_4_ and evaporated under reduced pressure. The products (**IV**) and (**V**) were purified by silica gel column chromatography (CH_2_Cl_2_/C_2_H_5_OH, 0–30%).

6-(Cholesteryloxycarbonylamino)-hexanoic acid (**IV**). Yield 1.12 g (82%). ^1^H-NMR (400 MHz, CDCl_3_, δ, ppm) (Supplementary Figure S1): 0.69 (s, 3H, H-18/19, Chol); 0.87 (d, 3H, H-26/27, Chol); 0.89 (d, 3H, H-26/27, Chol); 0.93 (d, 3H, H-21, Chol); 1.025 (s, 3H, H-18/19, Chol); 2.33 (t, 2H, -CH_2_-COOH); 3.35 (dd, 2H, -CH_2_-NH-,); 4.51 (m, 1H, H-3, Chol); 4.88 (t, 1H, -NH-,); 5.4 (d, 1H, H-6, Chol).

6-[2,5,7,6-Tetramethyl-2-(4′,8′,12′-trimethyltridecyl)-chroman-6-yloxycarbonyl]-hexanoic acid (**V**). Yield 1.17 g (80%). ^1^H-NMR (400 MHz, CDCl_3_, δ, ppm) (Supplementary Figure S2): 2.02 (s, 3H, -CH_3_, α-Toc); 2.06 (s, 3H, -CH_3_, α-Toc); 2.08 (s, 3H, -CH_3_, α-Toc); 2.37 (t, 2H, -CH_2_-COOH); 2.58 (t, 2H, 4,4-H, α-Toc); 3.27 (dd, 2H, -CH_2_-NH-); 5.09 (t, 1H, -NH-). ^13^C-NMR (100.6 MHz, CDCl_3_, δ, ppm): 179.0, 155.0, 149.0, 140.1, 127.5, 125.7, 122.7, 117.1, 74.9, 40.8, 39.9, 39.3, 37.3, 33.7, 32.6, 31.0, 29.6, 27.8, 26.0, 24.7, 24.3, 24.1, 23.8, 22.6, 20.9, 20.4, 19.5, 12.7, 11.8, 11.7. ESI-MS (*m*/*z*, C_36_H_61_NO_5_): [M-H]^−^ calcd.: 586.89, found: 586.50.

Methyl 6-(cholesteryloxycarbonylamino)-hexanoate (**VI**) and Methyl 6-[2,5,7,6-tetramethyl-2-(4′,8′,12′-trimethyltridecyl)-chroman-6-yloxycarbonyl]-hexanoate (**VII**) (Figure 2, step iv). Compound (**IV**) (1 g, 1.8 mmol) or compound (**V**) (1.1 g, 1.8 mmol) was dissolved in dry dichloromethane (15 mL), phosphorus trichloride (0.07 mL, 0.8 mmol) was added to the solution, and the reaction mixture was stirred for 16 h under argon at RT with the subsequent addition of absolute methanol (1 mL). The reaction was monitored by TLC (5% C_2_H_5_OH in CH_2_Cl_2_). The mixture was washed with water (100 mL), then with saturated aqueous NaHCO_3_ (100 mL), and twice with water (100 mL). The organic phase was dried under anhydrous Na_2_SO_4_ and evaporated to an oily residue. The products (**VI**) and (**VII**) were purified by silica gel column chromatography (CH_2_Cl_2_/C_2_H_5_OH, 0–2.5%).

Methyl-6-(cholesteryloxycarbonylamino)-hexanoate (**VI**). Yield 0.8 g (80%). ^1^H-NMR (400 MHz, CDCl_3_, δ, ppm) (Supplementary Figure S3): 0.69 (s, 3H, H-18/19, Chol); 0.87 (d, 3H, H-26/27, Chol); 0.89 (d, 3H, H-26/27, Chol); 0.93 (d, 3H, H-21, Chol); 1.02 (s, 3H, H-18/19, Chol); 2.29 (t, 2H, -CH_2_-C(O)OCH_3_,); 3.35 (dd, 2H, -CH_2_-NH-,); 3.65 (s, 3H, -CH_2_-C(O)OCH_3_,): 4.51 (m, 1H, H-3, Chol); 4.88 (t, 1H, -NH-,); 5.4 (d, 1H, H-6, Chol).

Methyl-6-[2,5,7,6-tetramethyl-2-(4′,8′,12′-trimethyltridecyl)-chroman-6-yloxycarbo nyl]- hexanoate (**VII**). Yield 0.97 g (90%). ^1^H-NMR (400 MHz, CDCl_3_, δ, ppm) (Supplementary Figure S4): 2.01 (s, 3H, -CH_3_, α-Toc); 2.05 (s, 3H, -CH_3_, α-Toc); 2.08 (s, 3H, -CH_3_, α-Toc); 2.33 (t, 2H, -CH_2_-C(O)OCH_3_); 2.58 (t, 2H, 4,4-H,α-Toc); 3.27 (dd, 2H, -CH_2_-NH-,); 3.67 (s, 3H, -CH_2_-C(O)OCH_3_); 5.06 (t, 1H, -NH-). ^13^C-NMR (100.6 MHz, CDCl_3_, δ, ppm): 173.9, 154.8, 149.1, 140.2, 127.5, 125.7, 122.7, 117.1, 74.9, 51.4, 40.8, 39.9, 39.2, 37.3, 33.8, 32.6, 31.0, 29.5, 27.9, 26.0, 24.7, 24.4, 23.8, 22.6, 20.9, 20.4, 19.6, 12.7, 11.7, 11.6. ESI-MS (*m*/*z*, C_37_H_63_NO_5_): [M+H]^+^ calcd.: 602.92, found: 601.10; [M+Na]^+^ calcd.: 624.92, found: 624.20; [M+2Na]^+2^ calcd.: 647.92, found: 647.40.

Hydrazide 6-(cholesteryloxycarbonylamino)-hexanoate (**VIII**) and Hydrazide 6-[2,5,7,6-tetramethyl-2-(4′,8′,12′-trimethyltridecyl)-chroman-6-yloxycarbonyl])-hexanoate (**IX**) (Figure 2, step *v*). Compound (**VI**) (0.5 g, 0.9 mmol) or compound (**VII**) (0.55 g, 0.9 mmol) was dissolved in methanol (5 mL). Hydrazine monohydrate (6 mL, 124 mmol) was added dropwise, and the reaction mixture was left for 8 h at RT. The reaction was monitored by TLC (15% C_2_H_5_OH in CH_2_Cl_2_). Compounds (**VIII**) and (**IX**) were precipitated in cold water (100 mL), the precipitates were filtered off, washed with water, and dried.

Hydrazide 6-(cholesteryloxycarbonylamino)-hexanoate (**VIII**). Yield 0.44 g (88%). ^1^H-NMR (400 MHz, CDCl_3_, δ, ppm) (Supplementary Figure S5): 0.69 (s, 3H, H-18/19, Chol); 0.87 (d, 3H, H-26/27, Chol); 0.89 (d, 3H, H-26/27, Chol); 0.93 (d, 3H, H-21, Chol); 1.025 (s, 3H, H-18/19, Chol); 2.13 (t, 2H, -CH_2_-C(O)NH-NH_2_,); 3.35 (dd, 2H, -CH_2_-NH-,); 4.51 (m, 1H, H-3, Chol); 4.88 (t, 1H, -NH-,); 5.4 (d, 1H, H-6, Chol); 6.75 (s, H, -CH_2_-C(O)NH-NH_2_).

Hydrazide 6-[2,5,7,6-tetramethyl-2-(4′,8′,12′-trimethyltridecyl)-chroman-6-yloxycarbonyl])-hexanoate (**IX**). Yield 0.35 g (64%). ^1^H-NMR (300 MHz, CDCl_3_, δ, ppm) (Supplementary Figure S6): 2.01 (s, 3H, -CH_3_, α-Toc); 2.05 (s, 3H, -CH_3_, α-Toc); 2.07 (s, 3H, -CH_3_, α-Toc); 2.58 (t, 2H, 4,4-H,α-Toc); 2.66 (t, 2H, -CH_2_-C(O)NH-NH_2_); 3.28 (dd, 2H, -CH_2_-NH-,); 5.08 (t, 1H, -NH-); 6.82 (s, H, -CH_2_-C(O)NH-NH_2_). ^13^C-NMR (125 MHz, CDCl_3_, δ, ppm): 173.4, 154.9, 149.0, 140.1, 127.4, 125.6, 122.7, 117.1, 74.8, 40.7, 39.9, 39.2, 37.3, 34.0, 32.6, 30.9, 29.5, 27.8, 26.0, 24.7, 24.5, 23.7, 22.5, 20.8, 20.4, 19.5, 12.7, 11.8, 11.6. ESI-MS (*m*/*z*, C_36_H_63_N_3_O_4_): [M+H]^+^ calcd.: 602.92, found: 602.10; [M+Na]^+^ calcd.: 624.92, found: 624.20.

### 4.3. Solid-Phase Synthesis of Polymer-Bound Oligonucleotides and Their 5′-Aldehyde-Containing Derivatives

Oligonucleotides dT_7_ and partially 2′-*O*-methyl-modified sense strand of siRNA targeted *MDR1* mRNA (siRNA/s), antireplicative RNAs (C57/1, C57/2, C57/3, 57/4, HB/1, HB/2), and guide RNAs for Mito-CRISPR/system (KSS/1, KSS/2, crRNA/1, crRNA/2, crRNA/3) were synthesized on an automatic DNA/RNA ASM-800 synthesizer (Biosset, Russia) at 0.4 µmol scale using solid-phase phosphoramidite synthesis protocols optimized for the instrument, with 8 min coupling step for 2′-*O*-TBDMS-protected ribophosphoramidites (0.1 M in acetonitrile), 6 min coupling step for 2′-*O*-methylated ribophosphoramidites (0.05 M in acetonitrile), 3 min coupling step for 2′-deoxythymidine phosphoramidites (0.05 M in acetonitrile), and 5-(ethylthio)-1H-tetrazole (0.25 M in acetonitrile) as an activating agent (sequences of oligonucleotides are shown in Table 1). Mixtures (*v/v*) of propionic anhydride/2,6-lutidine/tetrahydrofuran (10/10/80) and *N*-methylimidazole/tetrahydrofuran (16/84) were used as capping reagents. The oxidizing agent was 0.02 M iodine in pyridine/water/tetrahydrofuran (1/9/90). The detritylating reagent was dichloroacetic acid (3%) in dichloromethane. At the final step of the synthesis, 5′-Aldehyde-modifier C2 phosphoramidite (0.1 M in acetonitrile, 10 min coupling step) was employed. Modified polymer-bound oligonucleotides carrying a protected 5′-aldehyde group were used for the synthesis of lipophilic conjugates.

### 4.4. Optimization of the Solid-Phase Synthesis of Oligonucleotide Lipophilic Conjugates Containing Hydrazone Bond

(a) The model fully protected 5′-aldehyde-containing polymer-bound dT_7_ (20 mg), which was incubated in 80% acetic acid 1 h at RT to remove the acetal protecting group, and then, the polymer was washed with water (3 × 200 µL) and acetone (2 × 200 µL) and dried. To select the optimal conditions of conjugation, the obtained polymer-bound Ald-dT_7_ was divided into 9 portions (2 mg, about 0.06 µmol of the conjugate) with the addition of hydrazide of cholesterol (**VIII**) (0.35 mg, 0.6 µmol) and stirred for 12 h at RT at conditions, varying in the concentration of NaOAc buffer, pH 5.2 (0.1, 0.5, and 1 M) and the ratio of NaOAc buffer:dioxane (1:1, 1:3, 1:9). The total volume of each portion was 0.1 mL. Synthesized 5′-cholesterol-containing polymer-bound dT_7_ conjugate from each portion was washed with dioxane (3 × 200 µL), acetonitrile (2 × 200 µL), and acetone (2 × 200 µL) and then dried. The conjugate was deprotected and cleaved from the support by 0.05 M K_2_CO_3_ in methanol at RT for 16 h, neutralized with acetic acid, precipitated with 2% NaClO_4_ in acetone, and washed with acetone (see Section 4.4). The obtained samples were analyzed by 20% PAGE and visualized by Stains-all staining (Supplementary Figure S7).

(b) To study hydrazone bond stability under various deblocking conditions, we employed the control 5′-Chol-l-dT_7_ conjugate prepared by the solution-phase approach proposed by us earlier [48].

Several probes (each approx. 0.01 µmol) of the unprotected 5′-Chol-l-dT_7_ were processed under the following conditions [59,60,61]: (1) 400 μL of the 28% NH_3_ aqueous at 55 °C for 16 h; (2) 400 μL of the mixture 40% CH_3_NH_2_ aqueous/28% NH_3_ aqueous (1/1) (AMA-solution) at 65 °C for 15 min; (3) 400 µL of the AMA-solution at RT for 16 h; (4) 400 µL of the 0.05 M K_2_CO_3_ in methanol at RT for 16 h; and (5) 200 µL of *N*-methyl-2-pyrrolidone NMP/TEA^•^3HF/TEA mixture (150/100/75) at 65 °C for 1.5 h. At the end of the reaction, solutions (1)–(3) were evaporated to dryness; solution (4) was neutralized with acetic acid, precipitated with 2% NaClO_4_ in acetone, and washed with acetone; to the solution (5) ethoxytrimethylsilane was added to stop the reaction; the resulting precipitate was washed with acetone. The resulting dry residues were dissolved in 0.1 M NaOAc (pH 7.0), analyzed by 20% PAGE under denaturing conditions, and stained with Stains-all (Supplementary Figure S8).

### 4.5. Solid-Phase Synthesis of Oligoribonucleotide Lipophilic Conjugates Containing pH-Labile and Reduced Hydrazone Bond (***2–17***)

(a) Polymer-bound protected oligoribonucleotide carrying protected 5′-aldehyde-group (10 mg, 0.25–0.35 µmol) was incubated in 80% acetic acid for 1 h at RT to remove the acetal protecting group, washed with water (3 × 200 µL) and acetone (2 × 200 µL), and dried. For the conjugation, 50 µL of the solution of hydrazide of cholesterol (**VIII**) (1.9 mg, 3.5 µmol) or hydrazide of α-tocopherol (**IX**) (2.1 mg, 3.5 µmol) in dioxane was mixed with the same volume of 0.1 M NaOAc (pH 5.2) and added to the polymer-bound oligoribonucleotide, and then, this suspension was incubated 12 h at RT with stirring. Then, obtained polymer-bound protected conjugates (**2**–**4**, **6**, **8**–**17**) were washed with dioxane (3 × 200 µL), acetonitrile (2 × 200 µL), and acetone (2 × 200 µL) and dried.

(b) For the reduction in the hydrazone bond, to the suspension of polymer-bound cholesterol-containing conjugate (**3**) or (**5**) in a mixture of dioxane:NaOAc, 15 µL of 0.2 M NaBH_4_ in acetonitrile was added and incubated for 30 min at RT according to [62]. Then, the obtained polymer-bound conjugates (**5**, **7**) were washed with dioxane (3 × 200 µL), acetonitrile (2 × 200 µL), and acetone (2 × 200 µL) and dried.

(c) Protected polymer-bound oligonucleotide lipophilic conjugates (**2**–**17**) were cleaved from the support and deprotected by 0.05 M K_2_CO_3_ in methanol at RT for 16 h, neutralized with acetic acid, precipitated with 2% NaClO_4_ in acetone and washed with acetone. Further, 2′-*O*-Silyl groups were removed by the treatment with a mixture of NMP/TEA^•^3HF/TEA (150/100/75) at 65 °C for 1.5 h followed by ethoxytrimethylsilane addition to stopping the reaction. The resulting precipitates were washed with acetone and dried (Supplementary Figure S9). Deprotected lipophilic conjugates (**2**–**17**) were isolated by 12% denaturing PAGE, eluted from the gel with 0.3 M NaOAc (pH 7.0), and ethanol precipitated. All conjugates (**2**–**17**) (see Table 1) were homogeneous by the PAGE analysis data.

Aldehyde-modified oligoribonucleotide Ald-siRNA/s (siRNA/s: 5′-GGCUU^m^GAC^m^AAGUU^m^GU^m^AU^m^AU^m^GG-3′, Ald: C(O)H-C_6_H_4_-O-(CH_2_)_2_-O-P(O)(OH)-) was deprotected and isolated in the same manner.

Furthermore, 5′-Lipophilic conjugates of oligoribonucleotides (**2, 3, 5, 7**) and Ald-siRNA/s were characterized by ESI-MS analysis:

Ald-siRNA/s: theoretical mass 7066.34 Da, measured mass 7065.34 Da (Supplementary Figure S10);
Chol-l-siRNA/s (**2**): theoretical mass 7606.65 Da, measured mass 7603.70 Da (Supplementary Figure S11);
Toc-l-siRNA/s (**3**): theoretical mass 7650.70 Da, measured mass 7651.13 Da (Supplementary Figure S12);
Chol-l_1_-C57/1 (**5**): theoretical mass 13,390.19 Da, measured mass 13,391.36 Da (Supplementary Figure S13);
Chol-l_1_-C57/2 (**7**): theoretical mass 18,587.28 Da, measured mass 18,587.84 Da (Supplementary Figure S14).

### 4.6. Stability of the Hydrazone Bond of the Lipophilic Oligonucleotide Conjugates (***2***, ***3***) at Different pH

Chol-l-siRNA/s (**2**) or Toc-l-siRNA/s (**3**) (1 *A*_260_ unit) was incubated in Na_2_HPO_4_/KH_2_PO_4_ buffer (pH 4.8, 5.8, 6.8, or 7.8) at 37 °C for 1, 2, 3, 4, 5, and 24 h. The withdrawn aliquots (0.05 *A*_260_ units) were precipitated with 2% NaClO_4_ in acetone, washed with acetone, and products were analyzed by 12% denaturing PAGE and stained with ethidium bromide (Figure 4 and Figure 5; Supplementary Figure S15).

## 5. Conclusions

To conclude, we proposed the solid-phase approach allowing the efficient synthesis of 5′-lipophilic conjugates of oligonucleotides containing hydrazone bond. The developed method has the potential to be extended for the synthesis of pH-sensitive conjugates of oligonucleotides of various lengths and types (ribo-, deoxyribo-, 2′-*O*-methylribo-, and others) with ligands of different nature.

## Data Availability

Data are contained within the article and Supplementary Materials.

## Supplementary Materials

The following are available online, Figure S1: ^1^H NMR spectrum of (IV), Figure S2: ^1^H-NMR, ^13^C-NMR, and ESI-MS spectra of (**V**), Figure S3: ^1^H NMR spectrum of (VI), Figure S4: ^1^H-NMR, ^13^C-NMR, and ESI-MS spectra of (**VII**), Figure S5: ^1^H NMR spectrum of (VIII), Figure S6: ^1^H-NMR, ^13^C-NMR, and ESI-MS spectra of (**IX**), Figure S7: optimization of conditions of the solid-phase synthesis of 5′-Chol-l-dT_7_ (1), Figure S8: stability of hydrazone bond of 5′-Chol-L-dT_7_ (1) under treatment in different deblocking conditions, Figure S9: PAGE-analysis of reaction mixtures after the synthesis of conjugates (**2–4, 6, 8–17**) with hydrazone bond, Figure S10: ESI-MS spectrum of Ald-siRNA/s, Figure S11: ESI-MS spectra of Chol-l-siRNA/s and product of its destruction, Figure S12: ESI-MS spectra of Toc-l-siRNA/s and products of its destruction, Figure S13: ESI-MS spectrum of Chol-l_1_-C57/1, Figure S14: ESI-MS spectrum of Chol-l_1_-C57/2, Figure S15: Kinetic curves of hydrazone bond cleavage in lipophilic conjugates Chol-l-siRNA/s and Toc-l-siRNA/s at different pHs.

## Sample Availability

Samples of the compounds are not available from the authors.
