# Supplementary material for: A Versatile Solid-Phase Approach to the Synthesis of Oligonucleotide Conjugates with Biodegradable Hydrazone Linker
Source: Molecules. 2021 Apr 7;26(8):2119. doi: 10.3390/molecules26082119 (PMC8067880; doi:10.3390/molecules26082119)
Supplement: Supplementary file 1 [file molecules-26-02119-s001.pdf]

Electronic supporting information for

A versatile solid-phase approach to the synthesis of oligonucleotide conjugates with biodegradable hydrazone linker†

Mariya I. Meschaninova [1,*], Nina S. Entelis [2], Elena L. Chernolovskaya [1], Alya G. Venyaminova [1]

[1] Institute of Chemical Biology and Fundamental Medicine, Siberian Branch of Russian Academy of Sciences, Novosibirsk 630090, Russia; elena_ch@niboch.nsc.ru (E.L.C.); ven@niboch.nsc.ru (A.G.V)
[2] UMR Genetique Moleculaire, Genomique, Microbiologie (GMGM), Strasbourg University - CNRS, Strasbourg 67084, France; n.entelis@unistra.fr
*Correspondence: mesch@niboch.nsc.ru; Tel: +7-383-3635129

Content

	Page
**Figure S1.** ^{1}H-NMR spectrum of (**IV**)	**S2**
**Figure S2.** ^{1}H-NMR, ^{13}C-NMR and ESI-MS spectra of (**V**)	**S3**
**Figure S3.** ^{1}H-NMR spectrum of (**VI**)	**S4**
**Figure S4.** ^{1}H-NMR, ^{13}C-NMR and ESI-MS spectra of (**VII**)	**S5**
**Figure S5.** ^{1}H-NMR spectrum of (**VIII**)	**S6**
**Figure S6.** ^{1}H-NMR, ^{13}C-NMR and ESI-MS spectra of (**IX**)	**S7**
**Figure S7.** Optimization of conditions of the solid-phase synthesis of 5'-Chol-L-dT$_7$ (**1**)	**S8**
**Figure S8.** Stability of hydrazone bond of 5'-Chol-L-dT$_7$ (**1**) under treatment in different deblocking conditions	**S9**
**Figure S9.** PAGE-analysis of reaction mixtures after the synthesis of conjugates (**2-4,6,8-17**) with hydrazone bond	**S10**
**Figure S10.** ESI-MS spectrum of Ald-siRNA/s	**S11**
**Figure S11.** ESI-MS spectra of Chol-L-siRNA/s and product of its destruction	**S12**
**Figure S12.** ESI-MS spectra of Toc-L-siRNS/s and products of its destruction	**S13**
**Figure S13.** ESI-MS spectrum of Chol-L$_1$-C57/1	**S14**
**Figure S14.** ESI-MS spectrum of Chol-L$_1$-C57/2	**S15**
**Figure S15.** Kinetic curves of hydrazone bond cleavage in lipophilic conjugates Chol-L-siRNA/s and Toc-L-siRNA/s at different pH	**S16**

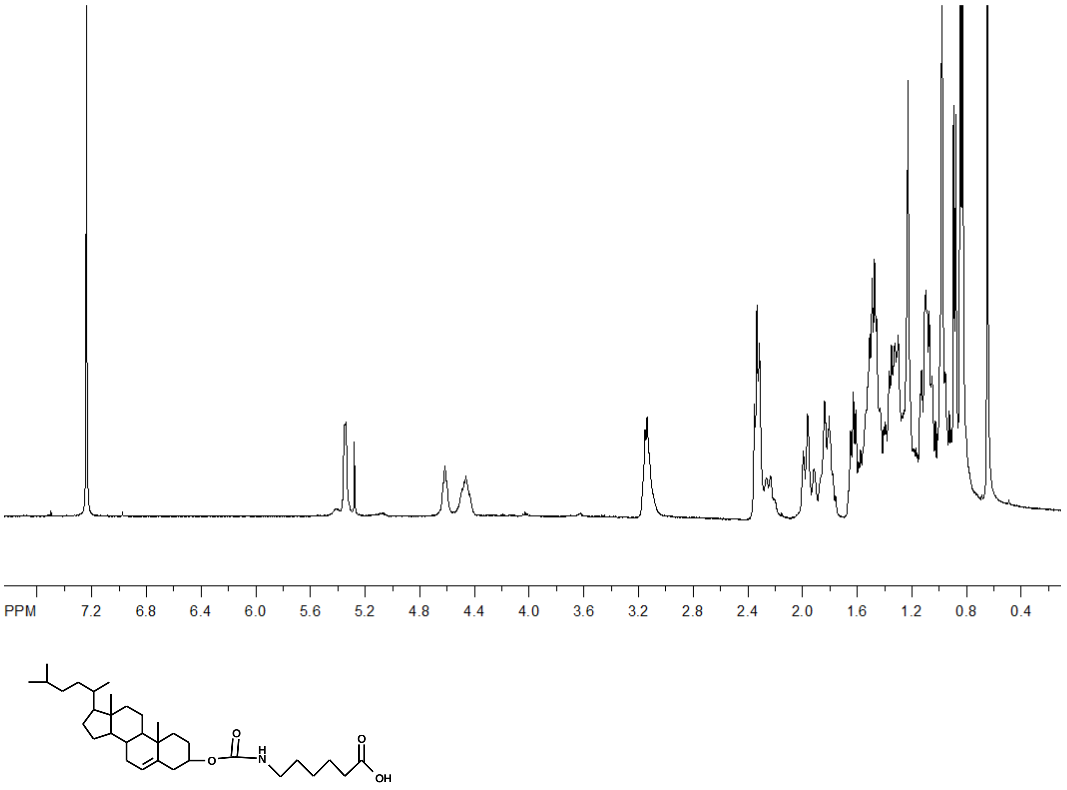

Figure S1. ^{1}H-NMR spectrum of 6-(cholesteryloxycarbonylamino)-hexanoic acid (**IV**). NMR spectrum was measured with $CDCl_3$ as a solvent using AVANCE III 400 NMR spectrometer. The assignment of peaks in the NMR spectrum is given in the experimental part.

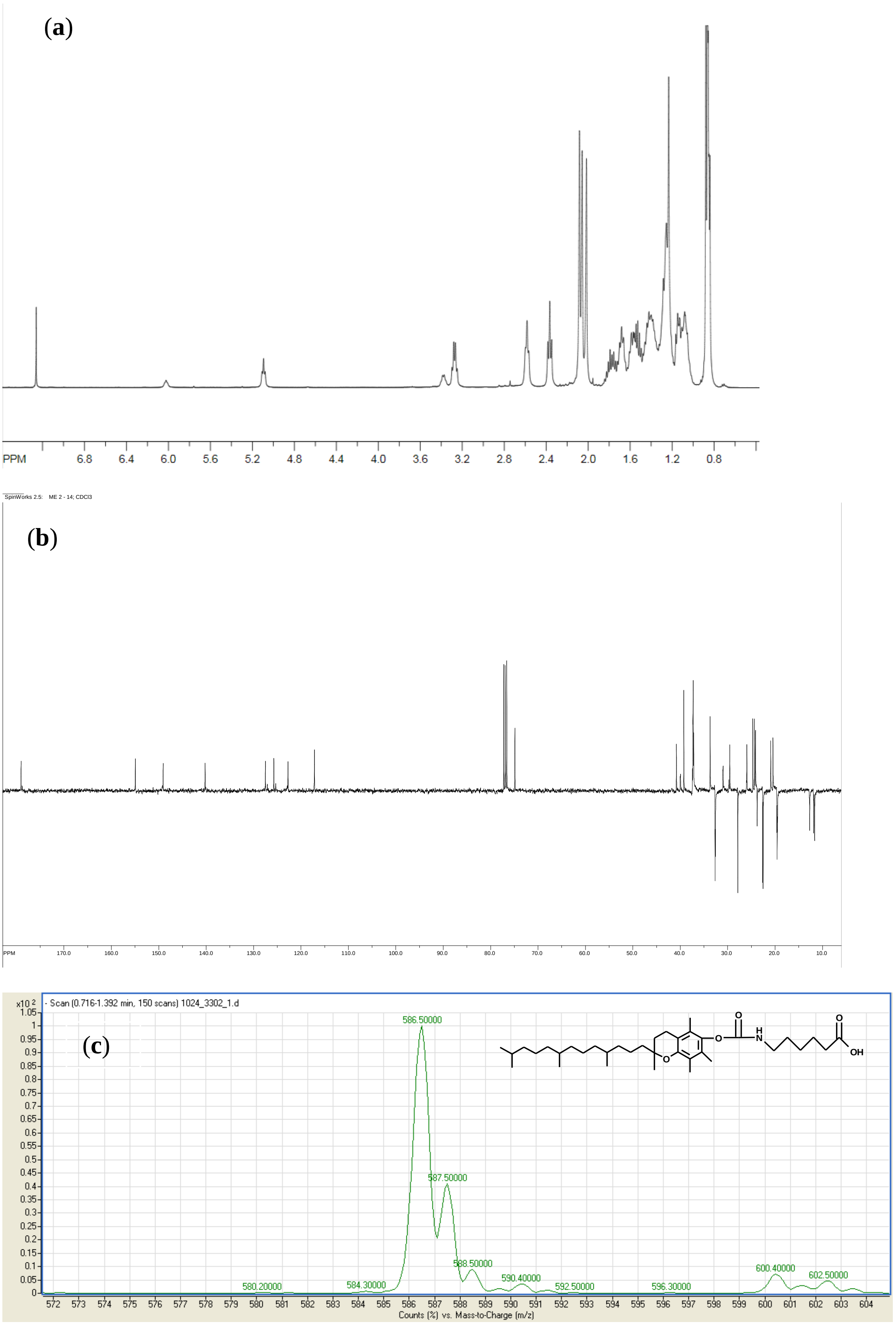

Figure S2. ^{1}H-NMR (**a**), ^{13}C-NMR (**b**) and ESI-MS (**c**) spectra of 6-[2,5,7,6-tetramethyl-2-(4',8',12'-trimethyltridecyl)-chroman-6-yloxycarbonyl]-hexanoic acid (**V**). NMR spectra were measured with $CDCl_3$ as a solvent using AVANCE III 400 NMR spectrometer. Mass spectrum was recorded by the ESI LC/MS XCT. The assignment of peaks in the NMR spectra is given in the experimental part.

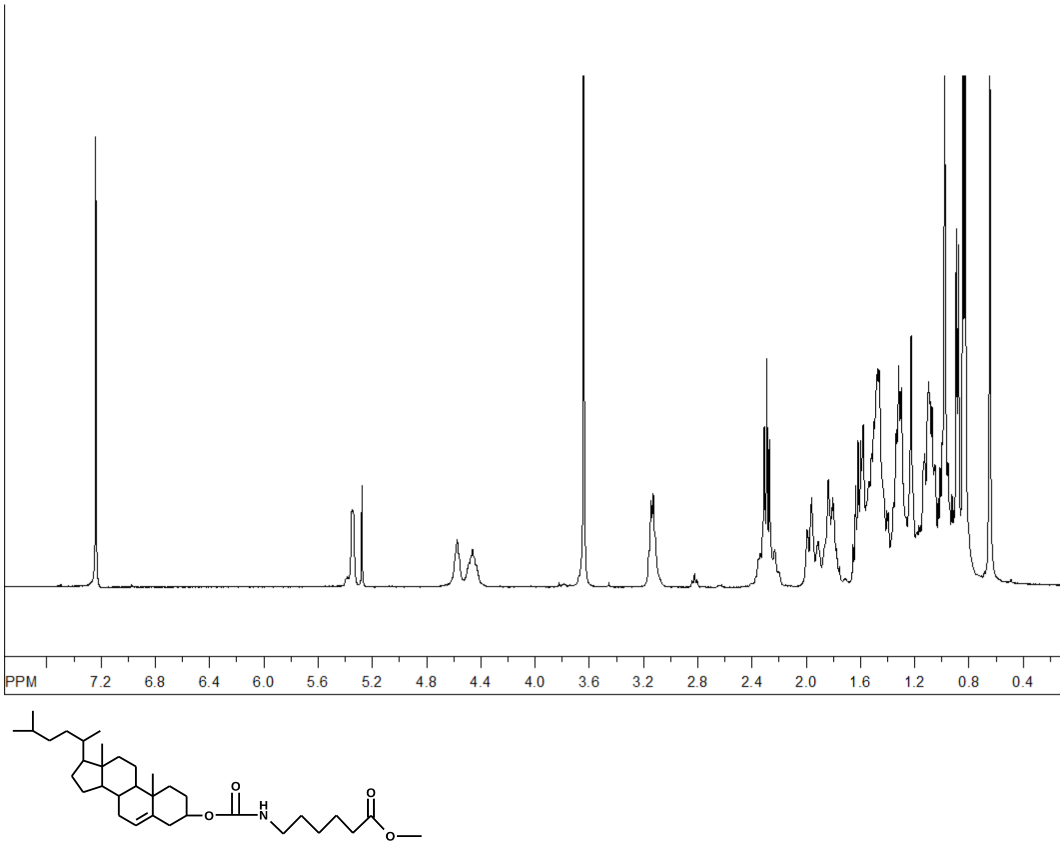

Figure S3. ^{1}H-NMR spectrum of methyl 6-(cholesteryloxycarbonylamino)-hexanoate (**VI**). NMR spectrum was measured with $CDCl_3$ as a solvent using AVANCE III 400 NMR spectrometer. The assignment of peaks in the NMR spectrum is given in the experimental part.

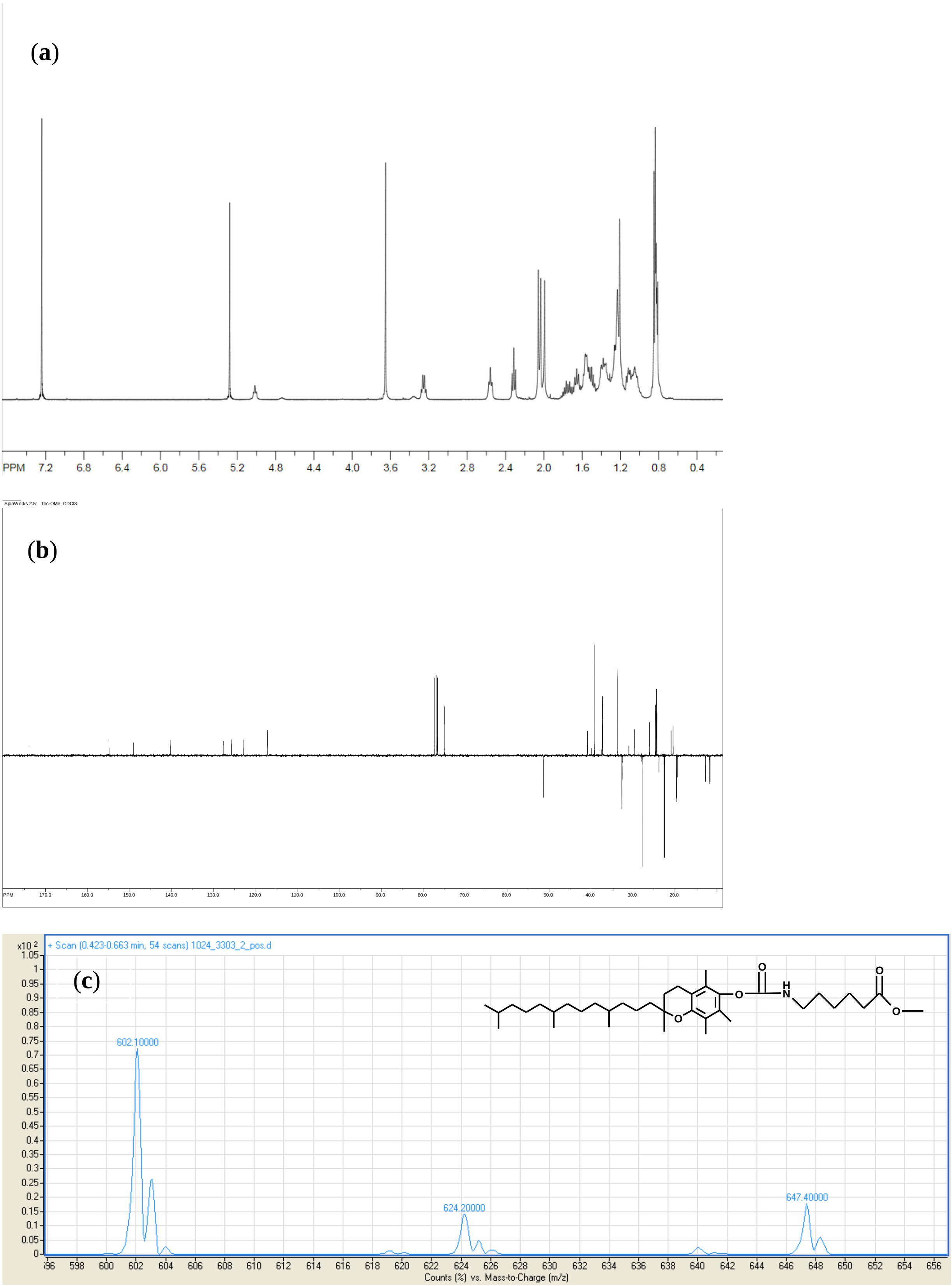

Figure S4. ^{1}H-NMR (**a**), ^{13}C-NMR (**b**) and ESI-MS (**c**) spectra of methyl 6-[2,5,7,6-tetramethyl-2-(4',8',12'-trimethyltridecyl)-chroman-6-yloxycarbonyl]-hexanoate (**VII**). NMR spectra were measured with $CDCl_3$ as a solvent using AVANCE III 400 NMR spectrometer. Mass spectrum was recorded by the ESI LC/MS XCT. The assignment of peaks in the NMR spectra is given in the experimental part.

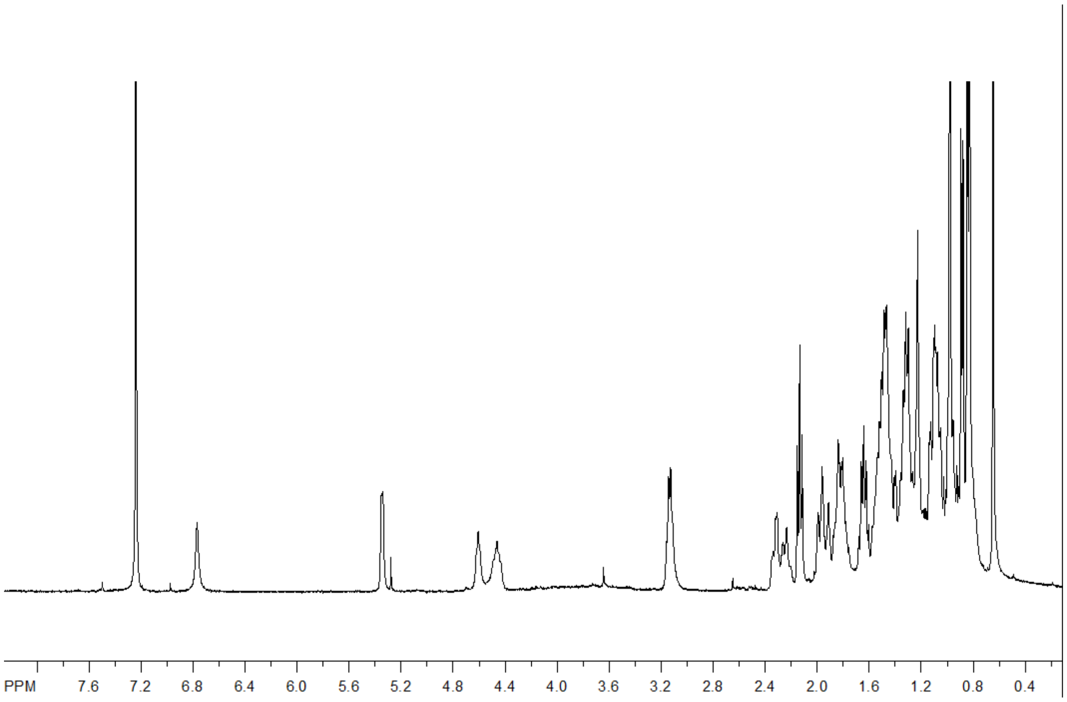

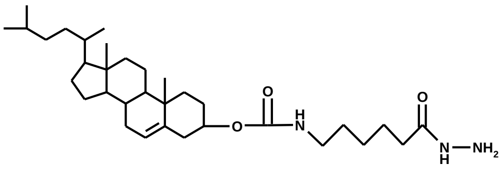

Figure S5. ^{1}H-NMR spectrum of hydrazide 6-(cholesteryloxycarbonylamino)-hexanoate (**VIII**). NMR spectrum was measured with $CDCl_3$ as a solvent using AVANCE III 400 NMR spectrometer. The assignment of peaks in the NMR spectrum is given in the experimental part.

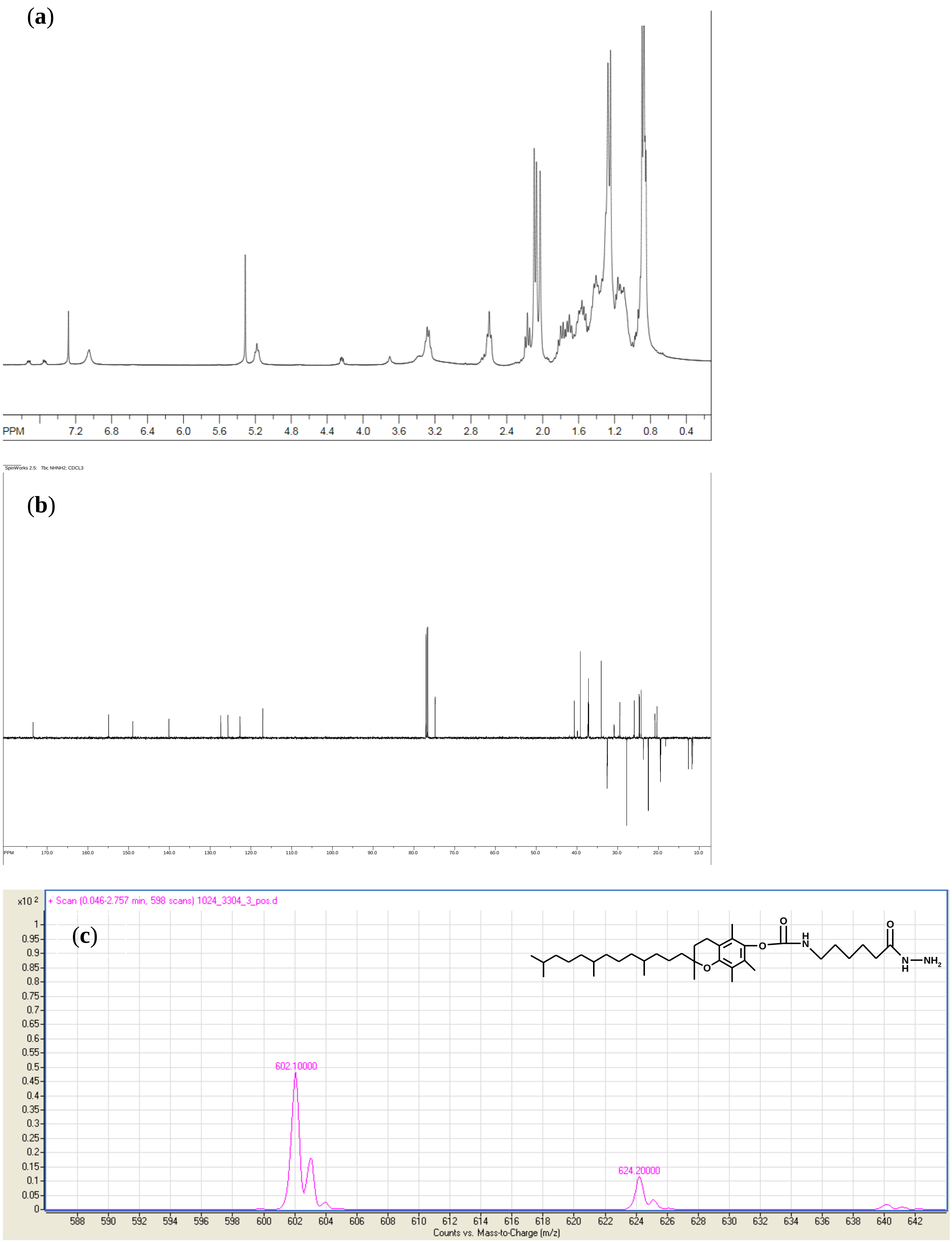

Figure S6. ^{1}H-NMR (**a**), ^{13}C-NMR (**b**) and ESI-MS (**c**) spectra of hydrazide 6-[2,5,7,6-tetramethyl-2-(4',8',12'-trimethyltridecyl)-chroman-6-yloxycarbonyl])-hexanoate (**IX**). NMR spectra were measured with $CDCl_3$ as a solvent using AVANCE III 400 and 500 NMR spectrometers. Mass spectrum was recorded by the ESI LC/MS XCT. The assignment of peaks in the NMR spectra is given in the experimental part.

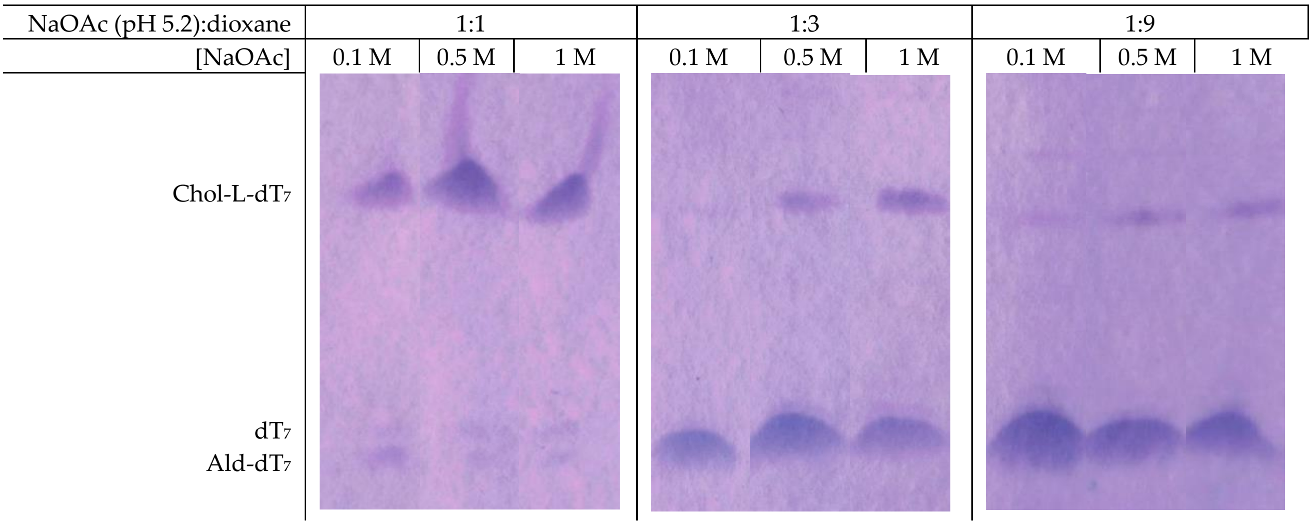

Figure S7. Optimization of conditions of the solid-phase synthesis of 5'-Chol-L-dT_7 (**1**). Chol: cholesterol residue; L: -OC(O)NH$(CH_2)_5$C(O)NH-N=CH-$C_6H_4O(CH_2)_2$OP(O)(OH)-; Ald: HC(O)$C_6H_4O(CH_2)_2$OP(O)(OH)-. Products were analyzed by gel electrophoresis in 20% PAAG under denaturing conditions (AA/bisAA 30:1, 7 M urea, TBE) and stained with Stains-all.

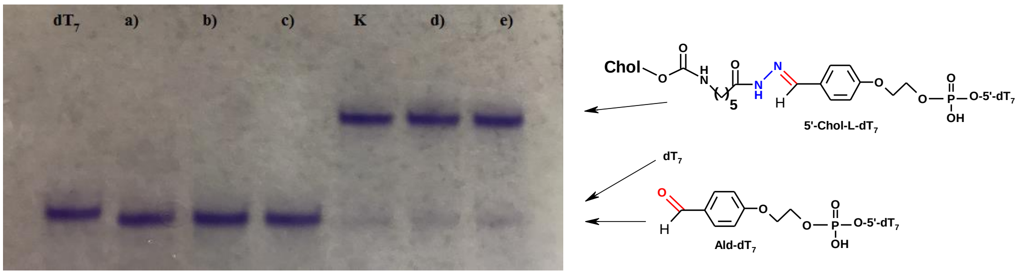

Figure S8. Stability of hydrazone bond of 5'-Chol-L-dT_7 (**1**) under treatment in different deblocking conditions: a) 28% NH_3 aq., 55 ^{O}C, 16 h; b) AMA-solution, 65 ^{O}C, 15 min; c) AMA-solution, RT, 16 h; d) 0.05M K_2CO_3 in methanol, RT, 16 h; e) NMP/TEA•3HF/TEA (150/100/75), 65 ^{O}C, 1.5 h. K: model 5'-Chol-L-dT_7; L: -OC(O)NH$(CH_2)_5$C(O)NH-N=CH-$C_6H_4O(CH_2)_2$OP(O)(OH)-; Chol: cholesterol residue. Products were analyzed by gel electrophoresis in 20% PAAG under denaturing conditions (AA/bisAA 30:1, 7 M urea, TBE) and stained with Stains-all.

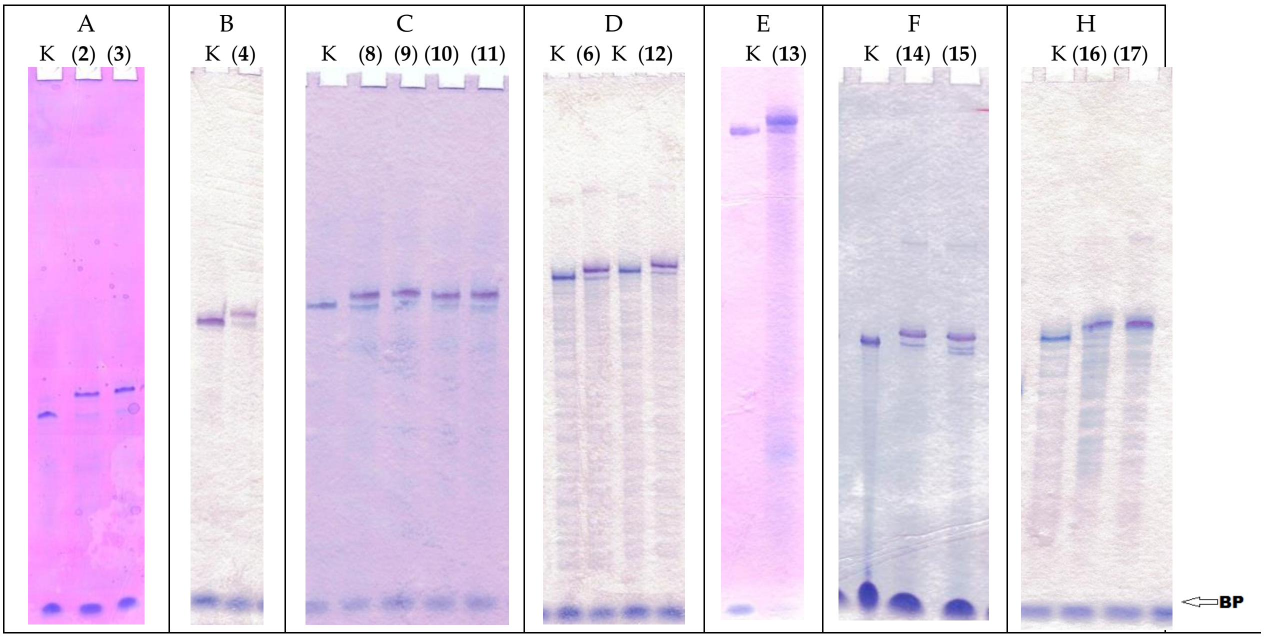

Figure S9. PAGE-analysis of reaction mixtures after the synthesis of conjugates (**2-4,6,8-17**) with hydrazone bond. (**A**) 5'-Lipophilic conjugates of sense strand of siRNA (**2,3**); (**B**) 5'-Lipophilic conjugate of mitochondrial antireplicative RNA (**4**); (**C**) 5'- Lipophilic conjugates of mitochondrial antireplicative RNAs (**8-11**); (**D**) 5'- Lipophilic conjugates of mitochondrial antireplicative RNA (**6**) and guide RNA (**12**); (**E**) 5'- Lipophilic conjugate of mitochondrial guide RNA (**13**); (**F**) 5'-Lipophilic conjugates of crRNAs (**14,15**); (**H**) 5'-Lipophilic conjugates of crRNAs (**16,17**). K – initial oligonucleotide. Structures of conjugates are given in Table 1. Products were analyzed by gel electrophoresis in 15% or 20% PAAG under denaturing conditions (AA/bisAA 30:1, 7 M urea, TBE) and stained with Stains-all. BP - bromophenol blue.

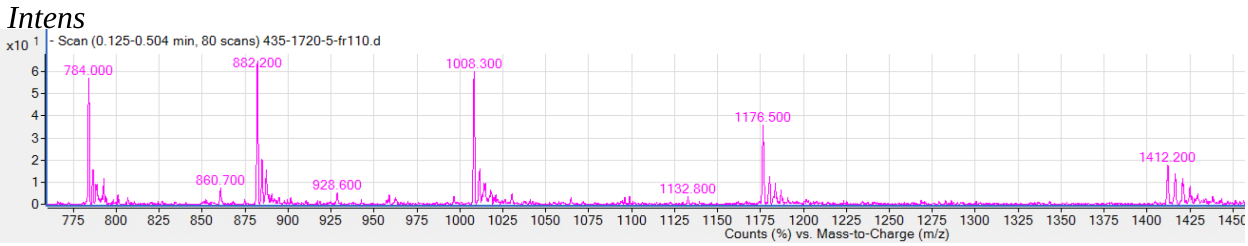

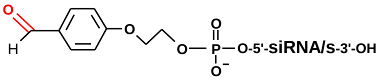

M_{calcd} 7066.34

[H9] / 784.00 M 7065.00

[H8] / 882.20 M 7065.60

[H7] / 1008.30 M 7065.10

[H6] / 1176.50 M 7065.00

[H5] / 1412.20 M 7066.00

M_{found} 7065.34

Figure S10. ESI-MS spectrum of Ald-siRNA/s. Ald: HC(O)$C_6H_4O(CH_2)_2OP(O)(OH)$-.

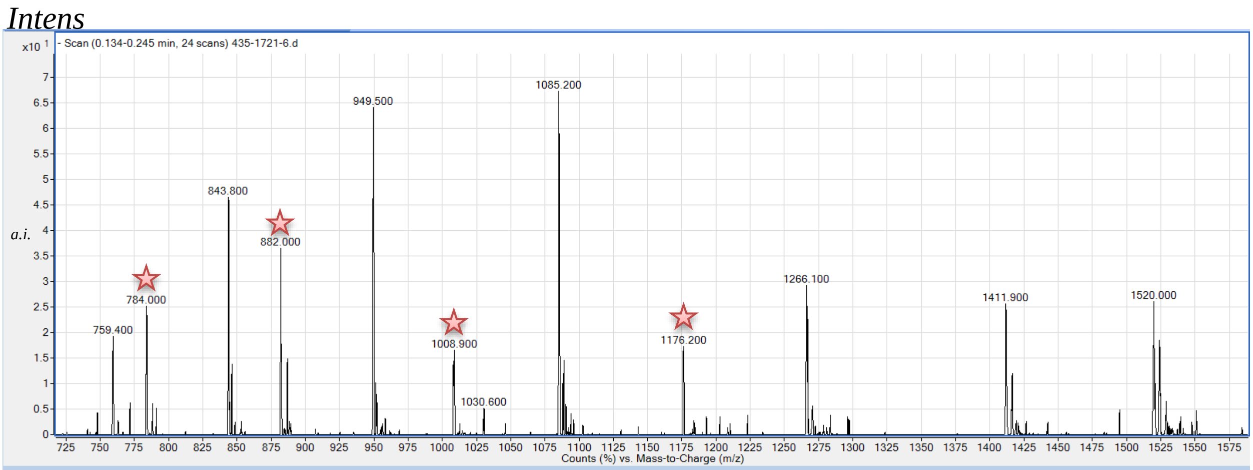

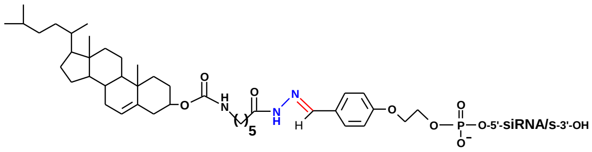

M_{calcd} 7606.65

[H10] / 759.40	M 7604.00
[H9] / 843.80	M 7603.20
[H8] / 949.50	M 7604.00
[H7] / 1085.20	M 7603.40
[H6] / 1266.10	M 7602.60
[H5] / 1520.00	M 7605.00

M_{found} 7603.70

Figure S11. ESI-MS spectra of Chol-L-siRNA/s and product (☆) of its destruction. Chol: cholesterol residue; L: - OC(O)NH$(CH_2)_5$C(O)NH-N=CHC_6H_4O$(CH_2)_2$OP(O)(OH)-.

☆- Aldehyde-containing oligonucleotide Ald-siRNA/s (see Figure S10).

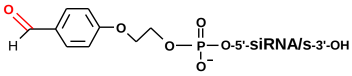

M_{calcd} 7066.34

[H9] / 784.00	M 7065.00
[H8] / 882.00	M 7064.00
[H7] / 1008.90	M 7069.30
[H6] / 1176.20	M 7063.20

M_{found} 7065.38

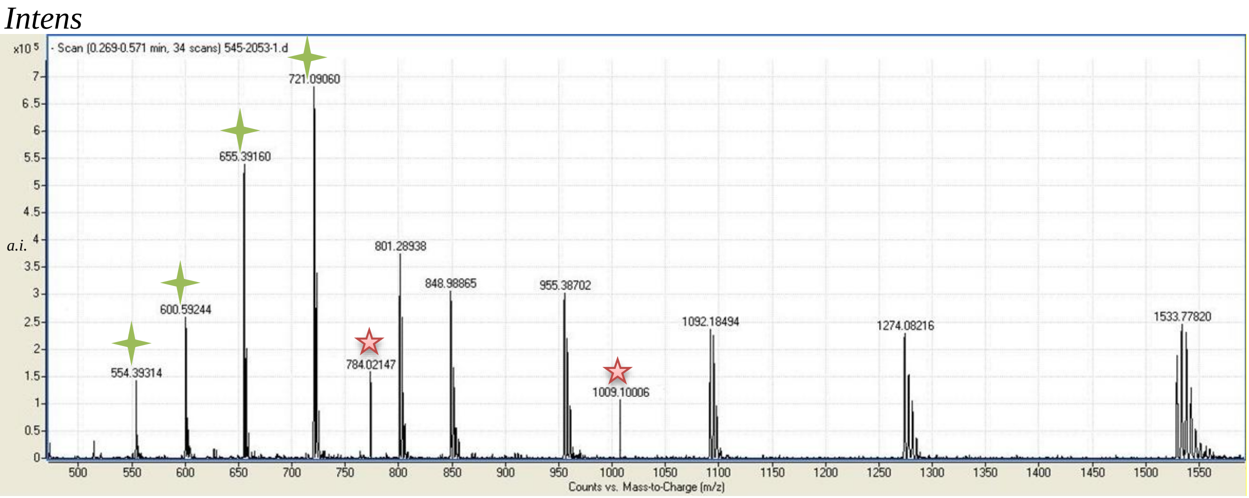

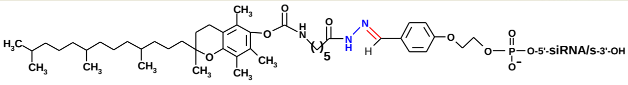

M

M_{calcd} 7650.7

[H9] / 848.99	M 7649.9
[H8] / 955.39	M 7651.1
[H7] / 1092.18	M 7652.29
[H6] / 1274.08	M 7650.49
[H5] / 1533.78	M 7673.89 – 23(M_{Na+}) + 1(M_{H+}) = M 7651.89

M_{found} 7651.13

Figure S12. ESI-MS spectra of Toc-L-siRNA/s and products (,) of its destruction. Toc: α-tocopherol residue; L: -OC(O)NH$(CH_2)_5$C(O)NH-N=$CHC_6H_4O(CH_2)_2$OP(O)(OH)-.

- Aldehyde-containing oligonucleotide Ald-siRNA/s (see Figure S10).

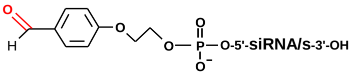

M_{calcd} 7066.34

[H9] / 784.02	M 7065.18
[H7] / 1009.10	M 7070.70

M_{found} 7067.94

- Isocyanate derivative of oligonucleotide siRNA/s.

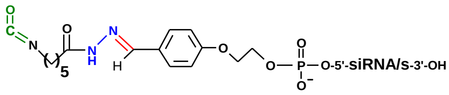

M_{calcd} 7221.00

[H13] / 554.39	M 7220.07
[H12] / 600.59	M 7219.08
[H11] / 655.39	M 7220.29
[H10] / 721.09	M 7220.9
[H9] / 801.29	M 7220.61

M_{found} 7220.19

Intens

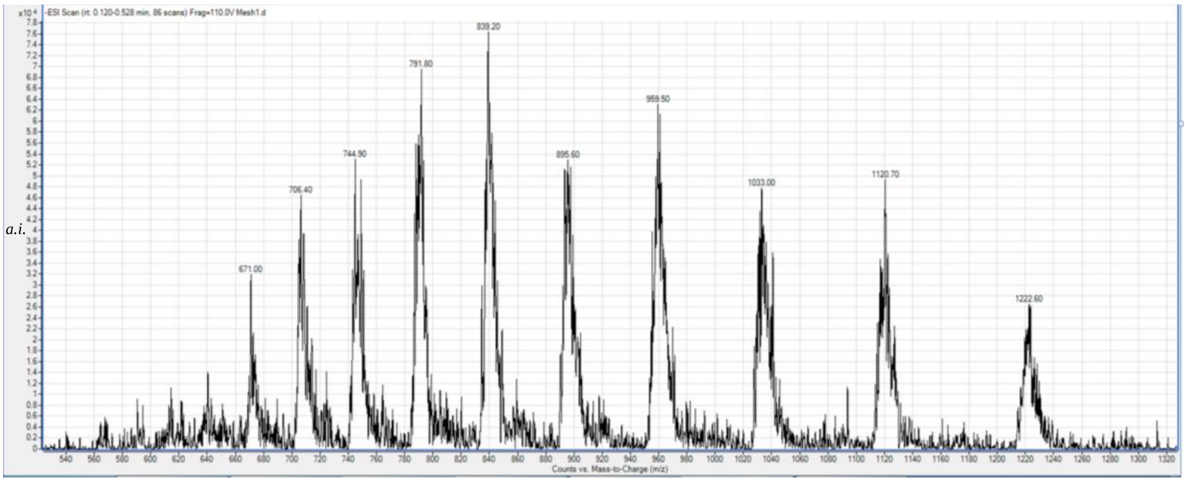

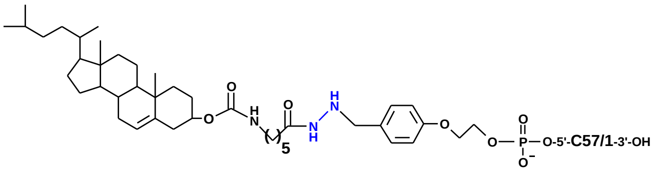

M_{calcd} 13390.19

[H20] / 671.00	M 13440.60 – 2*23(M_{Na+}) + 2*1(M_{H+}) = M 13396.00
[H19] / 706.40	M 13440.60 – 2*23(M_{Na+}) + 2*1(M_{H+}) = M 13396.60
[H18] / 744.90	M 13426.20 – 2*23(M_{Na+}) + 2*1(M_{H+}) = M 13382.20
[H17] / 791.80	M 13477.60 – 4*23(M_{Na+}) + 4*1(M_{H+}) = M 13389.60
[H16] / 839.20	M 13443.20 – 2*23(M_{Na+}) + 2*1(M_{H+}) = M 13399.20
[H15] / 895.60	M 13449.00 – 3*23(M_{Na+}) + 3*1(M_{H+}) = M 13383.00
[H14] / 959.50	M 13447.00 – 3*23(M_{Na+}) + 3*1(M_{H+}) = M 13381.00
[H13] / 1033.00	M 13442.00 – 2*23(M_{Na+}) + 2*1(M_{H+}) = M 13398.00
[H12] / 1120.70	M 13460.40 – 3*23(M_{Na+}) + 3*1(M_{H+}) = M 13394.40
[H11] / 1222.60	M 13459.60 – 3*23(M_{Na+}) + 3*1(M_{H+}) = M 13393.60

M_{found} 13391.16

Figure S13. ESI-MS spectrum of Chol-L_1-C57/1. Chol: cholesterol residue; L_1: -OC(O)NH(CH_2)$_5$C(O)NH-NH-$CH_2C_6H_4O(CH_2)_2$OP(O)(OH)-.

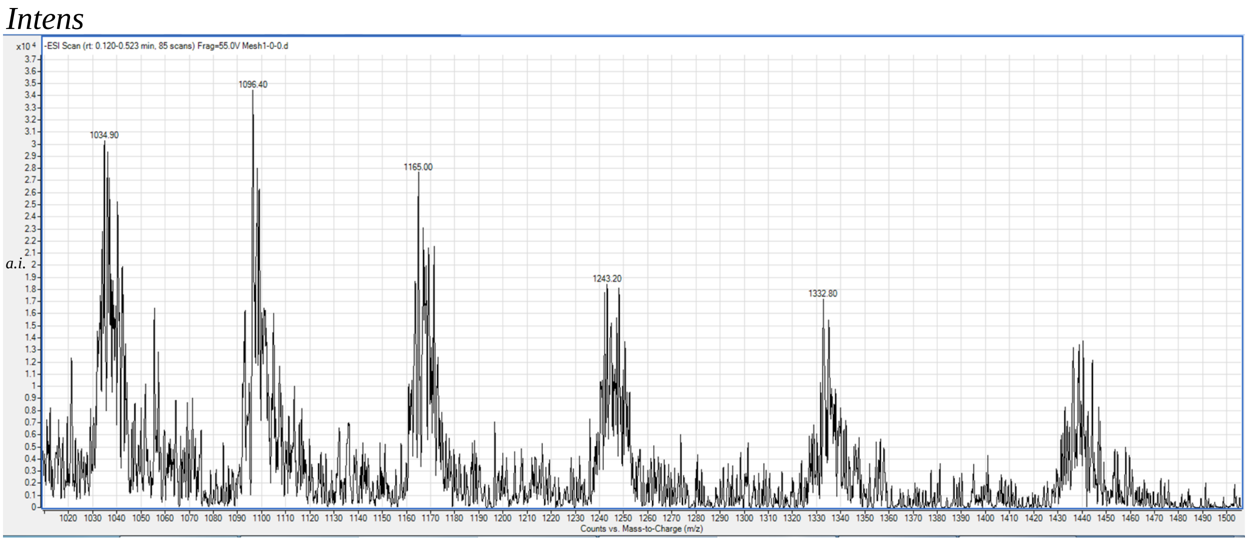

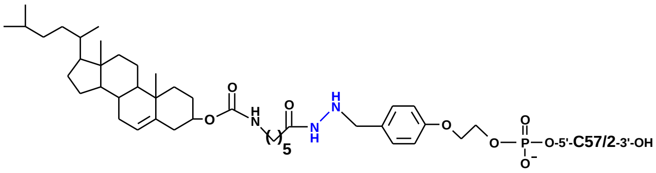

M_{calcd} 18587.28

[H18] / 1034.90	M 18646.20 – 3*23(M_{Na+}) + 3*1(M_{H+}) = M 18580.20
[H17] / 1096.40	M 18655.80 – 3*23(M_{Na+}) + 3*1(M_{H+}) = M 18589.80
[H16] / 1165.00	M 18656.00 – 3*23(M_{Na+}) + 3*1(M_{H+}) = M 18590.00
[H15] / 1243.20	M 18660.00 – 3*23(M_{Na+}) + 3*1(M_{H+}) = M 18594.00
[H14] / 1332.80	M 18673.20 – 4*23(M_{Na+}) + 4*1(M_{H+}) = M 18585.20

M_{found} 18587.86

Figure S14. ESI-MS spectrum of Chol-L_1-C57/2. Chol: cholesterol residue; L_1: -OC(O)NH(CH_2)$_5$C(O)NH-NH-$CH_2C_6H_4O(CH_2)_2$OP(O)(OH)-.

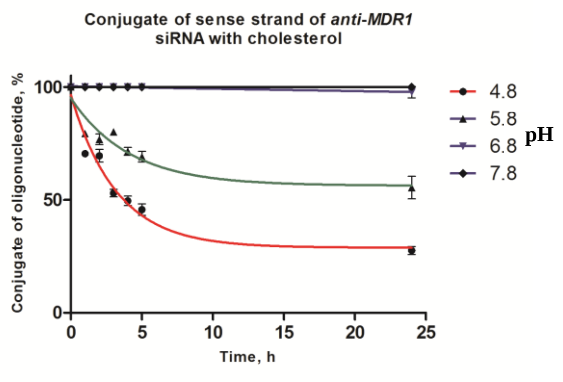

(**a**)

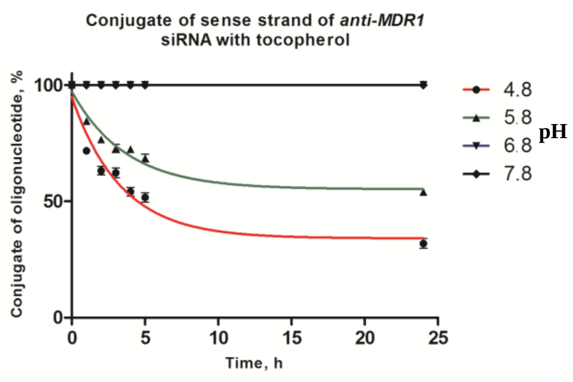

(**b**)

Figure S15. Kinetic curves of hydrazone bond cleavage in lipophilic conjugates Chol-L-siRNA/s (**a**) and Toc-L-siRNA/s (**b**) at different pH. Quantification of the full size conjugate (%, axis Y) depending on pH and the time of incubation. The results are mean value (±SD) from three independent experiments.
